# Plasmablastic lymphoma in Malawi

**DOI:** 10.1186/s13027-018-0195-4

**Published:** 2018-06-28

**Authors:** Takondwa Zuze, Matthew S. Painschab, Ryan Seguin, Evarista Kudowa, Bongani Kaimila, Edwards Kasonkanji, Tamiwe Tomoka, Bal Mukunda Dhungel, Maurice Mulenga, Maria Chikasema, Blessings Tewete, Asekanadziwa Ntangwanika, Sarah Chiyoyola, Fred Chimzimu, Coxcilly Kampani, Robert Krysiak, Nathan D. Montgomery, Yuri Fedoriw, Satish Gopal

**Affiliations:** 1 UNC Project-Malawi, Private Bag, A-104 Lilongwe, Malawi; 2 University of North Carolina, Chapel Hill, USA; 3 University of Malawi College of Medicine, Blantyre, Malawi; 4 Kamuzu Central Hospital, Lilongwe, Malawi

**Keywords:** Plasmablastic lymphoma, HIV, EBV, EPOCH, Sub-Saharan Africa

## Abstract

Plasmablastic lymphoma (PBL) clinical descriptions are scarce from sub-Saharan Africa (SSA) where both HIV and EBV are highly endemic. We identified 12 patients with pathologically confirmed PBL from a prospective cohort in Lilongwe, Malawi. Median age was 46 (range 26–71), seven (58%) were male, and six (50%) were HIV-positive. Eight patients were treated with CHOP and four with a modified EPOCH regimen. One-year overall survival was 56% (95% CI 24–79%), without clear differences based on HIV status. PBL occurs in Malawi in HIV-positive and HIV-negative individuals and can be treated successfully with curative intent, even in a low-resource setting in SSA.

## Introduction

Plasmablastic lymphoma (PBL) is an aggressive non-Hodgkin lymphoma (NHL) subtype associated with human immunodeficiency virus (HIV) infection [1], constituting 2% of all HIV-associated lymphomas [2]. Despite strong association with HIV, PBL is also seen in HIV-negative patients [3, 4]. Epstein-Barr virus (EBV) increases plasmablasts in blood and lymph nodes in the presence or absence of HIV, and may play a direct role in PBL development [5].

Despite high prevalence of HIV infection in sub-Saharan Africa (SSA), PBL descriptions from the region are scarce. We describe characteristics, treatment, and outcomes of PBL patients enrolled in a prospective, observational cohort in Malawi. This is among the first detailed clinical descriptions of this unique and aggressive NHL subtype from SSA, where HIV and EBV are endemic.

## Methods

We identified patients with PBL enrolled in the prospective Kamuzu Central Hospital Lymphoma Study between May 2013 and May 2017 in Lilongwe, Malawi. PBL diagnoses were histologically and immunophenotypically confirmed, and underwent subsequent secondary review in the United States [6]. All cases were positive for CD138 and negative for CD20 with Ki67 > 90%.

Following diagnosis, all patients underwent standardized clinical, laboratory, and radiologic assessments as previously described [7]. Before August 2016, patients were treated with CHOP (cyclophosphamide 750 mg/m^2^, doxorubicin 50 mg/m^2^, vincristine 1.4 mg/m^2^ day 1, and prednisone 40 mg/m^2^ days 1–5; 21 day cycles). After August 2016, patients received a modified EPOCH regimen (etoposide 50 mg/m^2^, vincristine 0.4 mg/m^2^, and doxorubicin 10 mg/m^2^ days 1–4, prednisone 60 mg/m^2^ days 1–5, and cyclophosphamide 750 mg/m^2^ day 1; 21-day cycles). Because the local clinical environment does not allow safe administration of 24-h continuous infusions, 24-h doses of etoposide, vincristine, and doxorubicin were instead administered over 6–8 h on successive days. Hematopoietic growth factors were not available. All HIV-infected patients received antiretroviral therapy (ART) concurrent with chemotherapy.

Cohort characteristics were summarized using simple descriptive statistics. Patients were followed until death or administrative censoring on September 30, 2017. Adverse events were reported using National Cancer Institute Common Terminology Criteria for Adverse Events version 4.0. Kaplan-Meier methods were used to estimate overall survival (OS). Analyses were conducted using Stata version 14.1 (College Station, Texas).

## Results

Of 183 adults with pathologically confirmed NHL during the study period, we identified 12 patients with PBL (Table 1). Median age was 46 years (range 26–71), seven (58%) were male, and six (50%) were HIV-positive. Of HIV-positive patients, three were diagnosed with HIV prior to PBL diagnosis, and had been on ART for 6, 10, and 14 months. Among HIV-positive patients, median CD4 cell count was 147 cell/μL (range 9–460) and three (50%) had suppressed HIV RNA < 400 copies/mL at PBL diagnosis. Of seven patients whose tumors were tested for EBV-encoded RNA (EBER) by in situ hybridization, five (71%) were positive: 3/3 HIV-positive, 2/4 HIV-negative. All patients had a primary site of disease in the head and neck. Nine (75%) patients had Ann Arbor stage I/II disease. Median lactate dehydrogenase was 339 IU/L (range 186–2522 IU/L, upper limit of normal 250 IU/L).

**Table 1:** Baseline characteristics of plasmablastic lymphoma patients from the Kamuzu Central Hospital Lymphoma Cohort study

	Total (*n* = 12)	HIV+ (*n* = 6)	HIV- (*n* = 6)
Age, years, median (range)	46 (26–71)	39 (30–60)	53 (26–71)
Male sex, n (%)	7 (58)	2 (33)	5 (83)
Largest tumor location head and neck, n (%)	12 (100)	6 (100)	6 (100)
CNS involvement, n (%)	1 (8)	0 (0)	1 (17)
Bone marrow involvement, n (%)	3 (25)	2 (33)	1 (17)
Largest diameter of mass, cm, median (range)	10 (3–22)	8 (3–15)	11 (10–22)
Ann Arbor stage I/II, n (%)	9 (75)	6 (100)	3 (50)
Performance score ≥ 2, n (%)	1 (8)	0 (0)	1 (17)
White blood cells, 10^3^/uL, median (range)	4.6 (2.7–14.9)	11.6 (6.2–12.5)	6.1 (2.7–14.9)
Absolute neutrophils, 10^3^/uL, median (range)	2.74 (0.92–10.6)	2.55 (0.9–4.07)	2.48 (1.3–10.6)
Hemoglobin, g/dL, median (range)	11 (6.2–14.9)	11.6 (6.2–12.5)	10.9 (10.1–14.9)
Platelets, 10^3^/uL, median (range)	206 (103–666)	210 (176–666)	233 (103–458)
Lactate dehydrogenase (LDH), IU/L, median (range)^a^	338 (186–2522)	328 (240–2522)	226 (186–1803)
CD4 count, cells/μL, median (range)	―	147 (9–460)	―
HIV RNA < 400 copies/mL, n (%)	―	3 (50)	―
EBER expression n (%)	5 (71)	3 (100)	2 (50)

*Abbreviations*: *CNS* Central nervous system, *EBV* Epstein Barr virus, *EBER* EBV encoded RNA, *ECOG PS* Eastern oncology cooperative group performance status, *HIV* human immunodeficiency virus, *LDH* lactate dehydrogenase

^a^LDH normal range = 0–250 IU/L

Eight patients were treated with CHOP for a median of six cycles (range 1–8). Four patients received the modified EPOCH regimen for a median of five cycles (range 2–6). All patients receiving EPOCH and 50% of CHOP recipients experienced treatment delay due to grade 3/4 neutropenia and one CHOP recipient experienced delay due to grade 3 anemia. Overall, five (42%) patients achieved a complete response (CR) after chemotherapy [CHOP 2/8 (25%); EPOCH 3/4 (75%)]. Both patients who achieved CR after CHOP relapsed 4 months after treatment completion.

**Fig. 1:**
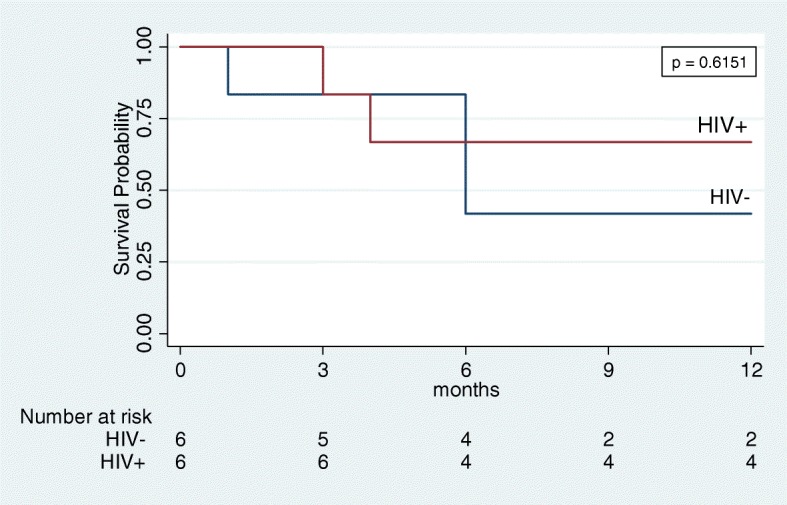
Overall survival of plasmablastic lymphoma patients in Lilongwe stratified by HIV status. No difference was found between HIV+ and HIV- patients by log-rank test

As of September 30, 2017, vital status was known for all patients and median follow-up was 13 months (range 1–35) among patients still alive. One-year OS was 56% (95% CI 24–79%, Fig. 1). No differences were observed between HIV-positive patients (1-year OS 67, 95% CI 19–90%) and HIV-negative patients (1-year OS 42, 95% CI 6–77%, *p* = 0.53). Of seven deaths, one was due to neutropenic sepsis and six due to lymphoma progression.

## Discussion

In resource-rich settings, PBL is a rare lymphoma subtype with poor outcomes [8, 9]. The current National Comprehensive Cancer Network guidelines recommend intensive chemotherapy regimens such as dose-adjusted EPOCH, hyper-CVAD (cyclophosphamide, vincristine, doxorubicin, dexamethasone alternating with cytarabine, methotrexate), or CODOX-M/IVAC (cyclophosphamide, vincristine, doxorubicin, methotrexate alternating with ifosfamide, etoposide, cytarabine). Consolidative high-dose chemotherapy and autologous stem cell rescue in first remission is also recommended by some [10]. Ongoing studies in high-income countries are attempting to investigate anti-myeloma agents (bortezomib, daratumumab, etc.) added to aggressive NHL chemotherapy regimens to improve outcomes, based on plasmacytic differentiation of PBL tumor cells [11, 12]. Conversely, other studies have demonstrated similar or worse results after intensive chemotherapy regimens compared with CHOP due to excess treatment-related complications [10, 13].

Upon first recognition of PBL in Malawi, and due to supportive care constraints, we initially treated participants with CHOP resulting in a 25% CR rate, with nearly all patients dying of progressive disease rather than treatment-related complications. Given these poor outcomes, we intensified our approach to a modified EPOCH regimen adapted as required by clinic infrastructure, resulting in a 75% CR rate, although follow-up time for this more recently treated group is limited. Nevertheless, this experience illustrates that even in a resource-limited country, sustained investments in cancer diagnostic and treatment capabilities can lead to recognition of new tumor types, foundational understanding of outcomes using standard approaches, and evidence-based adjustment of therapeutic approaches.

HIV-positive and HIV-negative patients with PBL have different clinical and pathologic characteristics, including better response to chemotherapy in HIV-positive patients [14]. HIV-positive PBL patients are younger, predominantly male, and have more frequent oral involvement. HIV-positive PBL cases have significantly higher expression of CD20, CD56, and EBER compared with HIV-negative PBL. HIV-positive patients with PBL have also been reported to have better survival than HIV-negative patients with PBL [14]. We found no difference in OS between HIV-positive and HIV-negative patients, although our sample size is small and trends in OS by HIV status were similar to those described in high-income countries.

Previous studies have indicated that PBL is closely associated with EBV infection. A metanalysis of PBL cases demonstrated EBV by in situ hybridization in 75% of HIV-infected cases and 50% of HIV-uninfected cases [9]. Similarly, our findings confirmed EBV presence in 3/3 HIV-positive cases but only 2/4 HIV-negative cases.

Strengths of our study include its prospective longitudinal nature, no loss to follow-up, standardized treatment, and pathologic confirmation using immunohistochemistry and telepathology consultation, all of which are not common in studies of lymphoproliferative disorders in SSA. Limitations are small sample size and relatively short follow-up. Ongoing enrollment and long-term follow-up will allow better understanding of prognostic factors and outcomes.

In conclusion, PBL occurs in SSA and may be underdiagnosed in many settings. Further study is needed to identify best treatment approaches, but our early experience suggests treatment intensification might lead to better outcomes for this population even in highly resource-limited environments.

## Abbreviations

CD4: Positive for cluster of differentiation 4
CHOP: Cyclophosphamide, doxorubicin, vincristine, and prednisone
EBER: Epstein-Barr virus-encoded RNA
EBV: Epstein Barr virus
EPOCH: Etoposide, prednisone, vincristine, cyclophosphamide and doxorubicin
HIV: Human immunodeficiency virus
OS: Overall survival
PBL: Plasmablastic lymphoma
